# Phylogenetic Placement and Taxonomy of the Genus Hederorkis (Orchidaceae)

**DOI:** 10.1371/journal.pone.0122306

**Published:** 2015-04-22

**Authors:** Joanna Mytnik-Ejsmont, Dariusz L. Szlachetko, Przemysław Baranow, Kevin Jolliffe, Marcin Górniak

**Affiliations:** 1 Department of Plant Taxonomy and Nature Conservation, The University of Gdansk, Wita Stwosza 59, PL-80-308, Gdańsk, Poland; 2 Cousine Island, Conservation Department, Seychelles; 3 Department of Molecular Evolution, The University of Gdansk, Wita Stwosza 59, PL-80-308, Gdańsk, Poland; Hellas, GREECE

## Abstract

Three plastid regions, *mat*K, *rpl*32-*trn*L and *rpl*16 intron and the ITS1-5.8S-ITS2 nuclear ribosomal DNA were used to demonstrate a phylogenetic placement of the genus *Hederorkis* (Orchidaceae) for the first time. The taxonomic position of this genus has been unclear thus far. The phylogenetic and morphological relations of *Hederorkis* to the most closely related genera *Sirhookera*, *Adrorhizon*, *Bromheadia* and *Polystachya* are also discussed. A hypothesis concerning an origin and evolution of *Hederorkis* is proposed. *Hederorkis* is an epiphytic two-leaved orchid genus with lateral inflorescence, non-resupinate flowers, elongate gynostemium and rudimentary column foot. It is native to the Indian Ocean Islands. Two species of *Hederorkis* are recognized worldwide, *H*. *scandens* endemic to Mauritius and Réunion and *H*. *seychellensis* endemic to Seychelles. For each of the species treated a full synonymy, detailed description and illustration are included. The distribution map and dichotomous keys to the species have also been provided.

## Introduction

*Hederorkis* Thouars is a climber known exclusively from the Seychelles Archipelago. Its name is derived from two words, Latin *hedera* (ivy) and Greek *orkis* (orchid) and refers to the scandent habit of the plant [1, 2]. There are two species classified within the genus, both being narrow endemics [3]. *Hederorkis seychellensis* Bosser is confined to Seychelles, Mahé, Silhouette and Aldabra, whereas *Hederorkis scandens* Thouars occurs more towards the south, in Mauritius and Réunion exclusively [4].

The two species have different ecological requirements, which is associated with different weather conditions. The Mascarenes, where *H*. *scandens* occurs, are sometimes subjected to very high levels of rainfall, up to 6,000 millimeters per year in La Réunion, in contrast, the Seychelles, where *H*. *seychellensis* grows, are relatively dry region with a lower altitude reaching only 914 millimeters at its highest in Mourne Seychellois National Park, however the climate in La Réunion can be dry on western slopes.

The genus was classified within the Polystachyinae Schltr. by Schlechter [5], Dressler [6, 7], Chase et al. [8], Szlachetko [9], and Cribb in Pridgeon et al. [10] because of its gynostemium structure as it produces a single tegula and viscidium, sticky caudicles and four subspherical pollinia. Morphologically the genus differs from the members of the subtribe in terms of its 2-leaved stems, lateral inflorescence, sepals and petals similar in shape and size, lack of mentum and rudimentary column foot. However, Cribb in Pridgeon et al. [10] also suggest possible relation of *Hederorkis* to *Adrorhizon*, *Bromheadia* and *Sirhookera* as the result of one of the analyses [10].

Senghas [11] transferred *Hederorkis* to his broadly defined subtribe Cymbidiinae Benth. along with genera such as, *Graphorkis* Thouars, *Grammatophyllum* Bl., *Cyrtopodium* R.Br., *Cryptarrhena* R.Br., and *Govenia* Lindl. Recently, Russell and Chase (unpublished, excerpted in [10]) suggested that *Hederorkis* is sister to *Polystachya*.

Due to lack of DNA material up till now, the taxonomic position of *Hederorkis* has been unclear and the further study was needed, especially at the molecular level. In this study, a taxonomic position is estimated using four DNA markers (plastid *mat*K, rpl32-trnL, rpl16 intron and nuclear ITS1-5.8S-ITS2). All of the regions have been used in the phylogenetic analyses in the previous studies of Orchidaceae. The *mat*K was used at the family [12] and generic [13] levels. The rpl32-trnL and rpl16 intron at the generic level [14, 15]. The nrDNA ITS regions have been used to infer phylogenetic relationships at tribal [16] and generic [17, 14] levels.

Due to the suitable variation of the *mat*K gene for analyses at the family level, this fragment was used as a reference marker. Based on the results obtained from the *mat*K gene sequences sampling was performed for others molecular markers.

## Material and Methods

### Morphological study

The study presented here is based on the examination of twelve herbarium specimens from two herbaria, K and P, acronyms according to Thiers [18], representing *Hederorkis*, including the types. These studies were supplemented by field work in Mourne Seychellois National Park in Mahé (Seychelles) conducted by one of the authors (KJ). A scientific expedition, approved by the Seychelles Bureau of Standards with the number of Research Approval Letter A0347, April 8th, 2010 and conducted by one of the authors (KJ). The aim of the visit to the Mourne Seychellois National Park in Mahé was to obtain the leaf fragments of *Hederorkis seychellensis* for the DNA analyses and to observe the specimens in its natural habitat. As the species is very rare (and usually also sterile in the field), it was not collected for the voucher to be deposited in the herbarium and a photo voucher was taken instead. The identification of *H*. *seychellensis* in the field was done accurately, as the species is very characteristic in its vegetative form and cannot be confused with other species in the Seychelles. The results of the two-year studies conducted in the field, the herbaria, and laboratory are presented in this paper. It was approved by the Seychelles Bureau of Standards It was carried out with an assistance of Katy Beaver from the Seychelles Plant Conservation Action group.

Standard procedure of preparing the herbarium material to facilitate stereomicroscopic observation was applied. The following vegetative organs and their characteristics of individual plants were analyzed: stem (height, shape, presence of glandular hairs), leaves (number, size, shape), sheaths (number, shape, size), inflorescence (size, density), floral bracts (size, shape, presence of glandular hairs) flowers, obtained from the middle part of the inflorescence (size of pedicel and ovary, presence of mentum, size and shape of lateral sepals, dorsal sepal, petals, and lip), as well as gynostemium (height and shape of column, presence of column foot). Particular parts of the flower were boiled, dissected, measured and drawn under a stereomicroscope. The results were then analyzed and compared with the type material, diagnoses and original illustrations. Drawings of some specimens were deposited in K. For all species, type material was available and examined.

### Plant material

DNA sequences for *mat*K were obtained from 219 taxa of Orchidaceae. Sequences were deposited in International Nucleotide Sequence Databases (INSD) under PopSet no 126789101 and 12678916 (S1 Annex). For *Bromheadia finlaysoniana* (GQ145085), *Bromheadia srilankensis* (GQ14086), *Adrorhizon purpurascens* (GQ145084), *Vanilla planifolia* (AJ310079), *Cypripedium calceolus* (AY557208), *Phalaenopsis aphrodite* (AY9164449), *Oncidium* Gower Ramsey (NC_014056) *Apostasia nuda* (AY557214), *Neuwiedia veratrifolia* (AY557211), *Hypoxis leptocarpa* (AY368375), *Rodohypoxis milloides* (AY368377), the *mat*K sequences were taken from INSD. For *rpl*32-trnL, *rpl*16 intron and ITS1-5.8S-ITS2 following sequences were taken from INSD: *Bromheadia srilankensis* (HQ222253/HQ222155/HM018544), *Bromheadia finlaysoniana* (HQ222254/HQ222154/GU556631), *Adrorhizon purpurascens* (HQ222257/HQ222153/GU556630), *Sirhookera lanceolata* (HQ222258/HQ222152/-), *Neobenthamia gracilis* (HQ222240/HQ222148/DQ091559), *Phalaenopsis aphrodite* (AY916449 from site 111963–112692/83492–84615/AY391542), *Oncidium* Gower Ramsey (NC_014056 from site 108560–109324/80997–79987/-), *Epipactis purpurata* (JN811741/JN811757/JN847416). Sequences *mat*K, *rpl*32-*trn*L, *rpl*16 intron and ITS1-5.8S-ITS2 for *Hederorkis seychellensis* were deposited in INSD under accession number KC339534/HQ222256/HQ222151/KJ401030—respectively. Data matrices are available as supporting material (S2 and S3 Annex).

#### DNA Isolation

Total genomic DNA was extracted from 20 mg of silica dried leaves [19] using the DNA Mini Plant Kit (A&A Biotechnology, Poland) following manufacturer protocol.

#### Amplification

ITS was amplified using primers: 17SE and 26SE [20]. The *mat*K was amplified with following two primers:- 19F [21] and 1326R [22]. The *rpl16* intron and *rpl*32*-trn*L intergenic spacer were amplified using primers developed by Shaw et al. [see [23,24] respectively]. The PCR mixture contained: dd H_2_O, 2.5 μl 10x Polymerase buffer with 15 mM MgCl_2,_ 1 μl of 5mM mix of each dNTP (200 μM), 0.5 μl of 10 mM primers, 1 μl MgCl_2_ (50 mM), 2.0 units of Taq DNA polymerase (EURx, Gdańsk, Poland) and genomic DNA. The thermal cycling protocol for *mat*K and ITS comprised 28 cycles, each with 45 s denaturation at 94°C, 45 s annealing at 52°C, an extension of 2 min 30 s/ 60 s for ITS at 72°C, concluding with an extension of 5 min at 72°C. The PCR cycling conditions for *rpl16* intron and *rpl*32-*trn*L were template denaturation at 80°C for 5 min followed by 30 cycles of denaturation at 95°C for 1 min, primer annealing at 50°C for 1 min, followed by primer extension at 65°C for 4 min. The final extension step was of 5 min at 65°C. Amplified products were cleaned with High Pure PCR Product Purification Kit (Roche Diagnostic GmbH, Mannheim, Germany) following manufacturer protocol.

#### Sequencing

Cycle sequencing was carried out directly on the purified product using Big Dye Terminator v 3.1 Cycle Sequencing Kit (Apllied Biosystems, Warrington, Cheshire, UK). Both strands were sequenced to assure accuracy in base calling. Sequence Navigator (Applied Biosystems) was used to edit the sequences and each individual base position was examined for agreement of the two strands using AutoAssembler (Applied Biosystems).

#### Phylogenetic analysis

Four data matrices were created for analyses. Large dataset for *mat*K containing 229 taxa representing the family Orchidaceae and the outgroup taxa. The small dataset for ITS contained 7 taxa, the small dataset for combined plastid analyses of *mat*K + rpl32-trnL + rpl16 intron contained 9 taxa, the fourth data matrix were created for the combined plastid and nuclear data (ITS). All DNA sequences were aligned by ClustalX [25] and adjusted by eye using Seaview [26]. All matrices were analyzed using heuristic search method of PAUP* (Phylogenetic Analysis Using Parsimony *and Other Methods) version 4.0b10 [27]. Optimality criterion was parsimony with tree-bisection-reconnection (TBR) branch swapping and the MULTREES option in effect, 1000 random addition replicates but keeping only 10 trees per replicate and ACCTRAN optimization. Gaps were treated as missing value. An internal support of the clades was evaluated by the bootstrap [28] with 500 replicates. All characters were unordered and equally weighted [29]. Consistency index (CI), retention index (RI), rescaled consistency index (RC) were calculated on one of the most parsimonious trees (MPT).

## Results

### Molecular data

**Fig 1:**
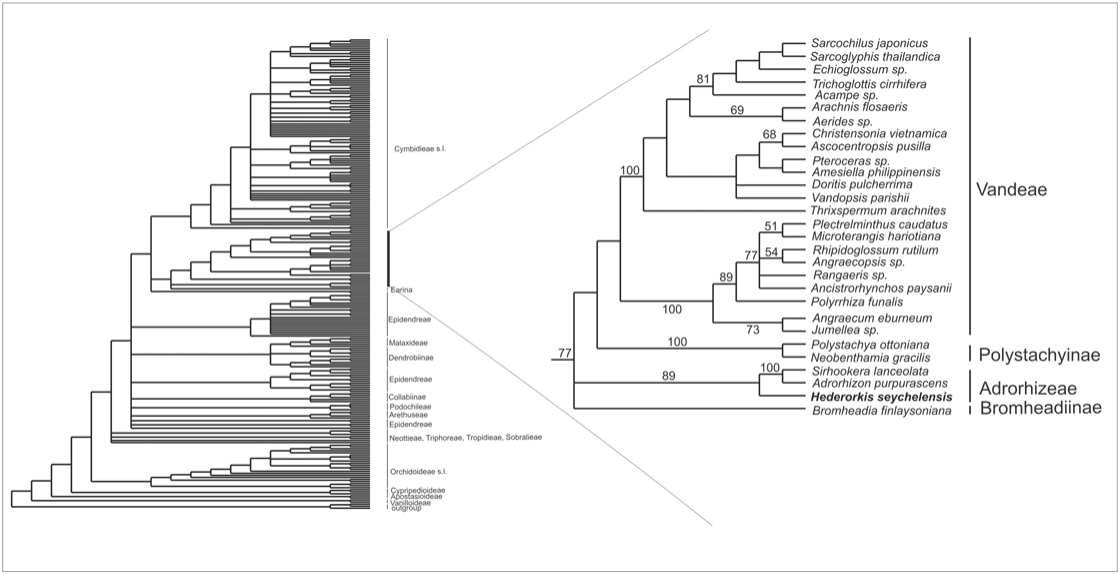
The strict consensus of 10.000 trees in *mat*K analysis (L = 4613, CI = 0.33 and RI = 0.68) for Orchidaceae. Bootstrap percentages >50 are given for supported clades above branches. Subfamilies, tribes and subtribes (sensu Chase et al. (8) with exception of Vandeae, Polystachyinae and Adrorhizeae) are indicated where applicable.

**Fig 2:**
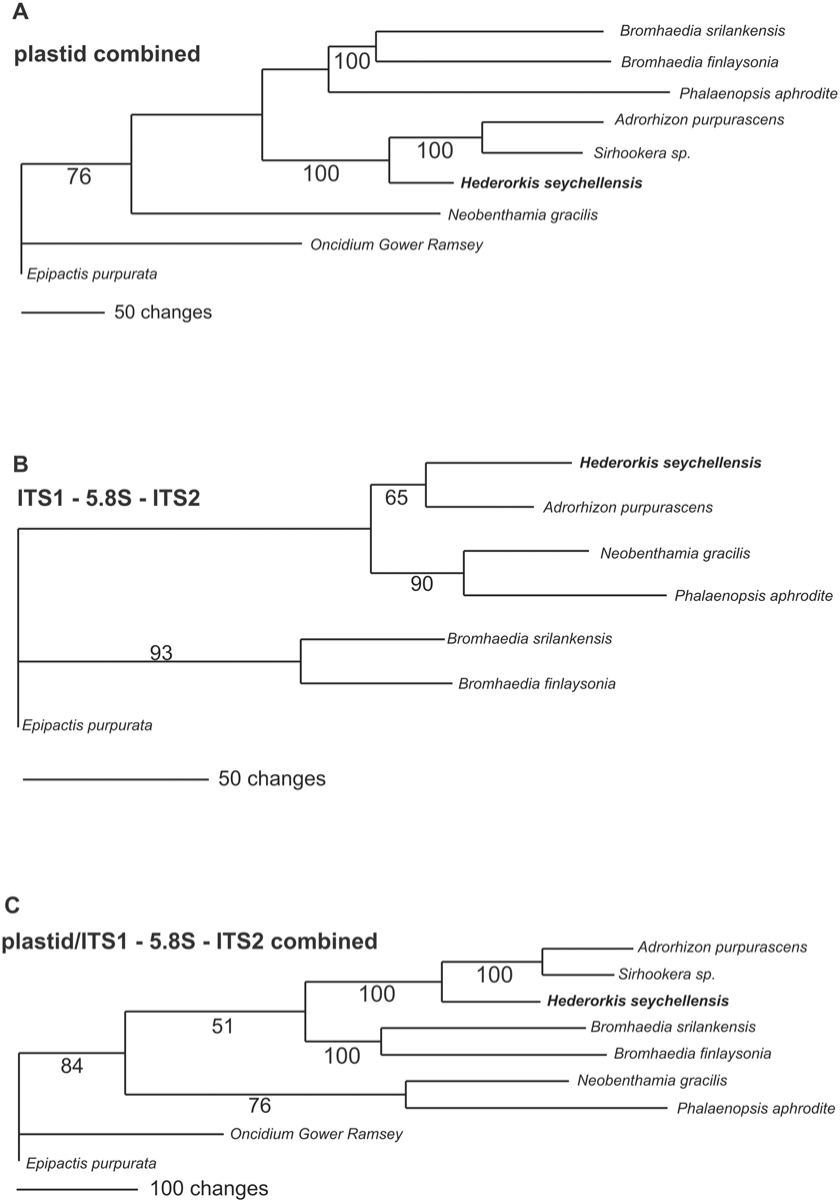
The most parsimonious tree (only one tree recovered in all parsimony analyses). Depicted as a phylogram to highlight the relative branch lengths in (A) plastid combined (matk + *rpl*32*-trn*L + rpl16 intron) analysis; (B) ITS1-5.8S-ITS2 analysis and (C) combined plastid and ITS1-5.8S-ITS2 analysis. *Hederorkis seychellensis* Thouars is highlighted in bold. The numbers above branches are Bootstrap percentages >50.

The statistics for all analyses are shown in Table 1. The strict consensus tree from the *mat*K analysis (large data set) is shown in Fig 1. *Hederorkis seychellensis* form a moderately supported clade (BS 77) along with Adrorhizeae (*Sirhookera lanceolata* and *Adrorhizon purpurascens*) and *Bromheadia finlaysoniana* + Polystachyinae + Vandeae. The above mentioned groups are successively sister to each other. The bootstrap (BS = 89) supports the *Hederorkis*-Adrorhizeae clade. The branch with *Bromheadia finlaysoniana* together with *Hederorkis* and Adrorhizeae (BS <50) collapses in the strict consensus tree. Adrorhizeae + *H*. *seychellensis* clade is also recovered by the analyses of other plastid regions, nuclear ITS sequences and combined analyses (Fig 2). All analyses (only small data set) recovered only one of the most parsimonious tree (Table 1). The bootstrap support for the Adrorhizeae + *Hederorkis seychellensis* clade is high (BS = 100) except for nuclear data (BS = 65). A visual comparison of topology shows an incongruence between the plastid and ITS data regarding to position of *Bromheadia* and *Neobenthamia* (Fig 2). On the plastid tree *Bromheadia* is sister to *Phalaenopsis aphrodite*; *Neobenthamia* is sister to all other taxa. However, on the ITS-based tree the position of the mentioned taxa is reversed.

**Table 1:** Statistics for one of the parsimonious trees from all analyses (CI, consistency index; RI, retention index; RC, rescaled consistency index).

Matrix	*mat*K	*matK+rpl*32-*trn*L + *rp*l16 intron	ITS1-5.8S-ITS2	Combined plastid +ITS1-5.8S-ITS2
**No. of taxa**	229	9	7	9
**Included positions in matrix**	1504	3588	637	4225
**Variable site**	922	812	236	1048
**Parsimony-informative sites**	676	206	91	297
**Trees (MPT)**	>10.000	1	1	1
**Fitch tree lenght**	4601	1099	354	1456
**CI**	0.3	0.8	0.8	0.8
**RI**	0.7	0.5	0.4	0.4
**RC**	0.2	0.4	0.3	0.4

### Taxonomic treatment and morphological data

#### *Hederorkis* Thouars

Nouv. Bull. Sci. Soc. Philom. Paris 19: 319. 1809. Type: *Hederorkis scandens* Thouars. An epiphytic or lithophytic, robust plant. Stem scandent, sympodial rhizomatous, covered with imbricate sheaths disintegrated into fibres, pseudobulbs rudimentary,. Leaves two, arising from the top of each shoot, elliptic, acute, divergent, thick, shortly articulated to a sheath. Inflorescence lateral simple raceme arising from a node on rhizome or near tip of shoot, few-flowered, longer than leaves. Flowers non-resupinate. Sepals and lateral petals free, somewhat similar, elliptic to oblanceolate, obtuse. No mentum. Lip ecallose, free to base, sparsely hairy on upper surface, obscurely trilobed, the lateral lobes erect, upcurved at the tips, the middle lobe porrect, larger than the lateral ones. Gynostemium elongate, slender, gently arched; column part free, much longer than the anther, narrowly winged, column foot rudimentary; anther incumbent, operculate, subglobose, slightly dorsiventrally compressed; connective narrow, rather thin; pollinia 4, in two pairs, obliquely superposed, unequal in size and form, oblong-ellipsoid, hard, caudiculae sticky, connecting pollinia with tegula; apical clinandrium obscure, collar-like; stigma elliptic, deeply concave; rostellum bent forwards, short, truncate; viscidium single, oblong-obovate, lamellar, delicate; tegula single, oblong, lamellar; rostellum deeply incised after removal of pollinarium.

**Fig 3:**
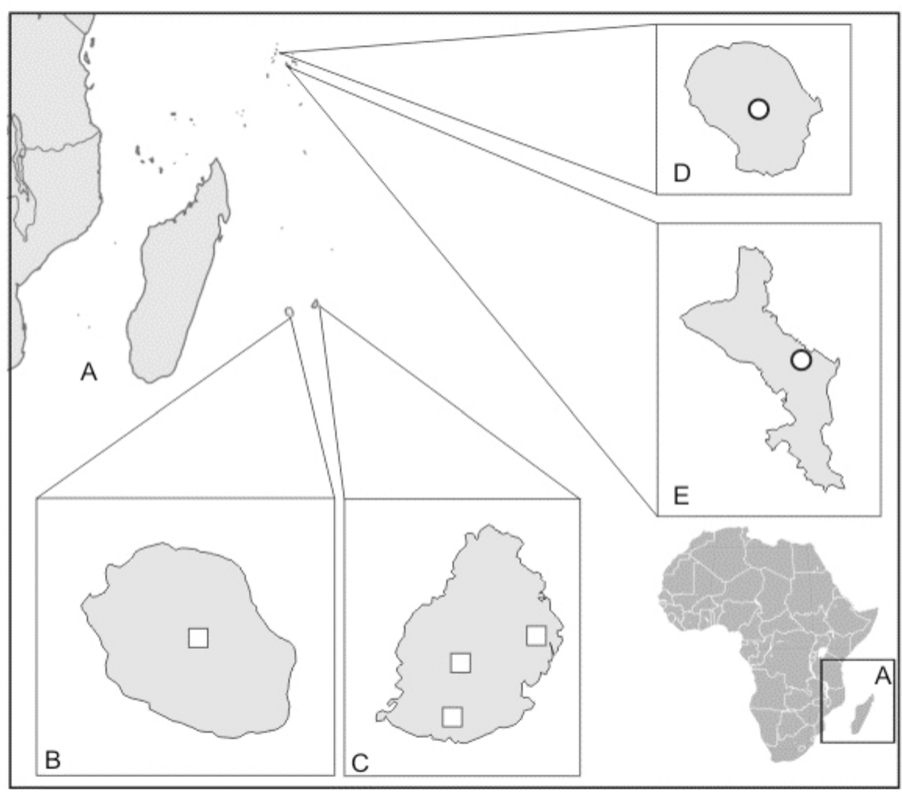
The distribution map of *Hederorkis scandens* Thouars (squares) and *H*. *seychellensis* Bosser (circles). A, East coast of Africa and Madagascar and neighboring islands. B, Réunion. C, Mauritius. D, Silhouette. E, Mahé.

Distribution. Mauritius, Réunion, Seychelles (Fig 3).

*Taxonomic note*. *Hederorkis*, described in 1809 by du Petit Thouars [30] has been considered to belong to *Bulbophyllum* (note in Kew Herbarium, according to Cribb in [10]) and Hunt [31]. However, Bosser [4] indicated closer relationship of the genus to *Polystachya* due to the gynostemium structure, and Dressler [6] agreed. Thouars used two set of names in his revolutionary system [30, 32], Rasmussen [33] clarified the nomenclature of *H*. *scandens*.

### A key to the species of *Hederorkis*

1 Leaves 4–6 x 1.3–2.5 cm, sepals 6–8 mm long, lip 5–6 x 2.5 mm, distinctly 3-lobed, ecallose ………. ***H*. *scandens***

1* Leaves 12–19 x 4.5–6.5 cm, sepals 10–12 mm long, lip 8–10 x 3 mm, obscurely 3-lobed, with two fleshy keels ............ ***H*. *seychellensis***

### *Hederorkis scandens* (Thouars) Bosser

Adansonia 16(2): 226. 1976. ≡ *Hederorkis* (as *Hederorchis*) *scandederis* (as "scaredederis") Thouars Hist. Orchid.: t. 91. 1822. ≡ *Neottia scandens* Thouars, Hist. Orchid.: t. 91. 1822, *non Bulbophyllum scandens* Rolfe, 1922. Holotype: Réunion, *Du Petit-Thouars 16* (P!) ≡ *Bulbophyllum mauritianum* P.F.Hunt, Kew Bull. 22: 491. 1968.

**Fig 4:**
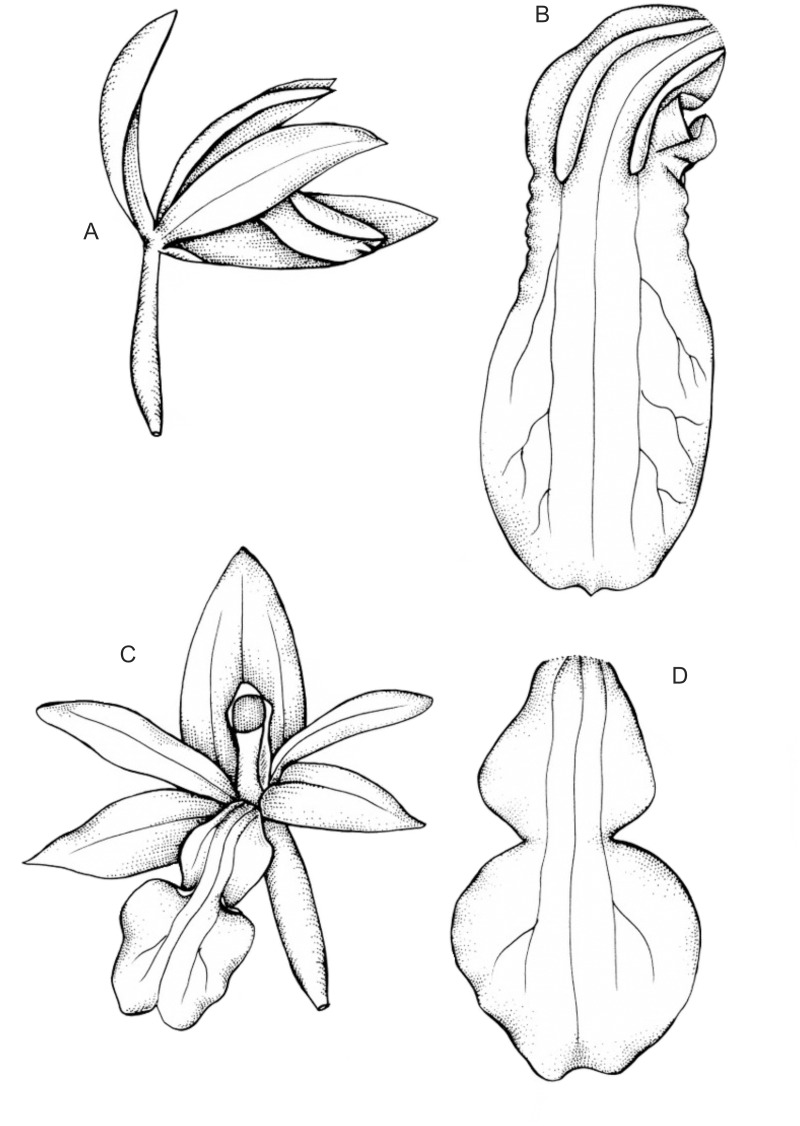
The drawings of the *Hederorkis* species. *Hederorkis scandens* Thouars (A—flower, B—lip) and *Hederorkis seychellensis* Bosser (C—flower, D—lip). Drawn by J. Mytnik-Ejsmont form the type specimens.

Epiphytic or litophytic plants. Stems not thickened basally, fusiform, erect, terminated with two opposite leaves, new shoot grows at the top of the previous one. Leaves 4–6 x 1.3–2.5 cm, elliptic, acute, thick, stout. Inflorescence 5–15 cm long, lateral, simple raceme, 3–6-flowered, pendent. Flowers non-resupinate, glabrous, purplish, relatively small. Pedicel and ovary 13 mm long. Floral bracts very small, up to 1 mm long, acute. Dorsal sepal 6–8 x 3 mm, oblong-elliptic, acute. Lateral sepals 6–8 x 2 mm, oblong-elliptic, acute. Petals 5–7 x 1.5 mm, ligulate, obtuse. Lip 5–6 x 2.5 mm, three-lobed, sessile; the lateral lobes 2 mm long, rounded; the middle lobe 3.2 x 2.5 mm, oblate, notched at apex. Gynostemium 4–4.5 mm high, column foot very obscure; anther hemispherical, 1–1.5 mm in diameter; viscidium semi-circular, 0.3–0.4 mm in diameter. Pollinia 4, ovoid, 0.6–0.7 mm long (Fig 4).

Distribution. Mauritius, Réunion.

Elevation. Up to 600 m.

Ecology. Epiphyte in forests, climbing rocks and trees.

Flowering. March-September.

Representative specimens. LA RÈUNION: *Du Petit-Thouars 16* (P!). MAURITIUS: Reserve de Perrier, 30 Mar 1934, *Vaughan 977* (K!, MAU); Ibid., *Vaughan 1411* (MAU); Bassin Blanc, *Guého & Staub 12627* (MAU); Ibid., Horne *s*.*n*. (K!); Entre le Mt. Camisard et le Piton Bombou, *Guého 14869* (MAU); Sine loc., *Justice Blackburn s*.*n*. (K!).

### *Hederorkis seychellensis* Bosser

Adansonia 16(2): 226. 1976. ≡ *Bulbophyllum scandens* Rolfe, Bull. Misc. Inform. Kew: 23. 1922, *non Hederorkis scandens* Thouars, 1822, *non Bulbophyllum scandens* Kraenzl., 1904. Lectotype (designated by Bosser 1976): Seychelles: Cascade Patates, *Thomasset 32* (K!).

An epiphytic or lithophytic robust plant. Stems 4-angled, thickened basally into small pseudobulbs, fusiform, erect, terminated with two opposite leaves, new shoot grows at the top of the previous one, internodes 1.5–2 cm long. Leaves 12–19 x 4.5–6.5 cm, oblong-elliptic, acute to subobtuse, subscarious. Inflorescence lateral; scape 10 cm long, arcuate; raceme up to 30-flowered. Flowers non-resupinate, glabrous, white, yellowish, cream, bright pink or purple. Pedicel and ovary 5–8.3 mm long, glabrous. Floral bracts 2 mm long, ovate, apiculate. Dorsal sepal 11–12 mm long, oblong, obtuse. Lateral sepals 10 x 3 mm, falcate, obtuse, fleshy at apices. Petals 9 x 2.5 mm, falcate, obtuse. Lip 8–10 x 3 mm, obscurely three-lobed, recurved, fleshy along the mid-nerve, the margins undulate, the basal third of the lip furnished with two glabrous elevated, fleshy and parallel 3.2 mm long keels; the lateral lobes very obscure; the middle lobe 5 x 3.5 mm, ligulate and flat, rounded at apex with a short apiculus, papillose, tessellated, undulate margins in basal part of the middle lobe. Gynostemium 5 mm high; column foot absent; anther hemispherical, 1 mm in diameter, viscidium 0.5–0.6 mm long, ovate, rounded at the apex; tegula 0.8 mm long (Fig 4).

Distribution. Endemic to the Republic of Seychelles (Mahé and Silhouette).

Elevation. 400–600 m.

Ecology. Epiphyte or lithophyte in montane forests, climbing rocks and trees, among mosses.

Flowering. May-September.

Representative specimens. SEYCHELLES: Mahé, Cascade Patates, May 1902, elev. 1400 ft., *Thomasset 32* (K!); Mahé, 1908, *Stanley Gardiner s*.*n*. (K!); Mahé, alt. 2000 ft., Sep 1960, *Archer 173* (K!); Mahé, Casse les Dents, 27 Sep 1906, *Dupont s*.*n*. (K!); Mahé, Frere mountain, elev. 500 m, *Jolliffe s*.*n*. (UGDA!—photo); Mahé et Silhouette, *Horne 603* (K!); Silhouette, Mare aux Cochon Sealark Expedition, 1908, *Stanley Gardiner s*.*n*. (K!); Doubtful material probably not representing the genus: Aldabra, Aug 1916, *Sine coll*., *s*.*n*. (K!).

Both species of *Hederorkis* are plants with similar habit and floral structure; however, *Hederorkis seychellensis* is a larger, more robust plant with an oblong, fleshy lip adorned by two longitudinal calli in the basal third. The lip of *H*. *scandens* is smaller, deeply constricted in the middle, widest in apical half and free from any callosities. *Hederorkis seychellensis* is uncommon in Mahé and very rare in Silhouette and probably does not occur in Aldabra. The material collected in Aldabra atoll is sterile and may not represent this species. *H*. *scandens* is known from four localities in Mauritius and one from Réunion. The islands on which the species occur are small and well known botanically, but *Hederorkis* have been reported from very few localities. The range of distribution of both species of *Hederorkis* coincide with an area of extremely high floral diversity and endemism (Fig 3), the Madagascar and the Indian Ocean Islands hotspot. The hotspot is one of the world’s top conservation priority due to its remarkable biodiversity and extensive deforestation. A potential distribution range for both species is relatively small, the known extent of occurrence of *H*. *seychellensis* is about 170 km^2^ and about 4560 km^2^ of *H*. *scandens*. Moreover, the islands are residential and strongly deforested, so the extent of potential habitats for *Heredorkis* is quite limited.

## Discussion

Prevalent usage of molecular methods in taxonomy throws new light on phylogenetic position of many taxa. Based on analyses of *mat*K sequences, *Hederorkis* appears to be closely related to Indian *Sirhookera* Kuntze and *Adrorhizon* Hook.f. *in* Trimen, as it is seen in the strict consensus tree. Both genera are embedded in the clade embracing i.e. Polystachyeae Pfitz. and Vandeae Lindl., with paleotropical, mostly Asian genera.

The taxonomic position of *Adrorhizon* and *Sirhookera* has varied. Both genera were classified either in Coelogyneae Lindl. [6], Dendrobieae Endl. [7], Epidendreae Humb., Bonpl. & Kunth [34, 7], Glomereae [35] or Vandeae Lindl. [10]. Based on morphological and anatomical characteristics Szlachetko [9] proposed a higher, tribal rank for Adrorhizinae. The presence of the *Calanthe*-type of velamen and clavate pollinia with caudiculae, according to Szlachetko [9], may indicate a relationship of Adrorhizinae with Bletieae Benth. and Podochileae Benth. & Hook. The *Vanda*-type of seed and presence of tegula found in *Adrorhizon* and *Sirhookera*, on the contrary, may suggest that both genera present one of the blind-lines of pre-vandoid orchids. The presence of tegula does not preclude certain connections with Coelogyneae Pfitz. We recapitulate the differences and similarities between *Hederorkis* and *Polystachya*, *Adrorhizon* and *Sirhookera* (genera to which it supposed to be related) in Table 2.

**Table 2:** The comparison of the morphological characters of *Hederorkis* Thouars, *Polystachya* Hook., *Sirhookera* Kuntze and *Adrorhizon* Hook.f. *in* Trim.

	*Hederorkis*	*Polystachya*	*Sirhookera*	*Adrorhizon*
**Pseudobulbs**	homoblastic	homoblastic	heteroblastic	heteroblastic
**Leaves**	two, apical, opposite, subpetiolate	1-few, apical, sessile to subpetiolate	single, petiolate	single, subsessile
**Inflorescence**	basal, few-flowered	apical, few-several-flowered	basal, branching, several-flowered	basal, few flowered
**Flowers**	nonresupinate	nonresupinate	resupinate	horizontal
**Lip**	3-lobed	3-lobed, occasionally simple	3-lobed	simple
**Callus**	two keels or missing	oblong	inconspicupus	no
**Mentum**	no	prominent	no	no
**Gynostemium**	elongate, slender, gently arched	short to elongate, rather slender, erect to gently arched	erect, slender, gently swollen towards the apex, rather delicate	slender, erect, rather delicate
**Column foot**	rudimentary	usually stout, prominent, as long as the column part, occasionally much shorter	vestigial	very short, but present, free at the apex
**Anther**	incumbent, operculate, subglobose, slightly dorsiventrally compressed	incumbent, operculate, conical, ovoid to transversely ellipsoid, 2-chambered, the inner anther partitions reduced	set above the stigma base, incumbent, dorsiventrally flattened, more or less cordate in outline, easily falling off, obscurely 4-chambered	positioned well above the stigma base, incumbent, dorsiventrally flattened, semi-ovoid, easily falling off, 4-chambered, chambers oblique
**Pollinia**	4, in two pairs, obliquely superposed, unequal in size and form, oblong-ellipsoid, hard	4, in two pairs, obliquely superposed, subequal in size and shape, oblong, elliptic to ovate in outline, hard	4, unequal in size and shape, superposed, obliquely clavate	4, in two pairs, semi-superposed, unequal in size and form, oblong to obliquely ovoid, flattened, attenuate towards the apex, easily becoming disintegrated
**Caudiculae**	sticky	sticky	produced from the apical part of each pollinium, sticky, granular	formed of apical part of each pollinium, sticky, granular
**Stigma**	elliptic, deeply concave	elliptic to transversely elliptic, deeply concave	large, cordate in outline, deeply concave	large, obtriangular in outline, deeply concave
**Rostellum**	bent forwards, short, truncate	more or less bent forward, short, ligulate, blunt to elongate, triangular, acute	ligulate, truncate, bent forwards, rather fleshy	ligulate, blunt, bent over the stigmatic surface, rather fleshy
**Viscidium**	single, oblong-obovate, lamellar, delicate	single, elliptic, transversely elliptic to obovate, lamellar	single, oblong, thin, membranous, delicate	single, elliptic, membranous, very delicate
**Tegula**	single, oblong, lamellar	single, oblong, lamellar	double, linear, delicate or probably absent	single, oblong-linear, thin, membranous, very delicate
**Rostellum remnant**	deeply incised	more or less notched at the apex	furcate, both lobes rather thick, fleshy	deeply incised

As can be seen *Hederorkis* shares some characters with *Polystachya* (homoblastic pseudobulbs, non-resupinate flowers) and some others with *Adrorhizon/Sirhookera* (obscure column foot). One of the interesting characters of *Hederorkis* are bifoliate pseudobulbs. We suppose it can be a synapomorphous state for the genus. Despite the similarities mentioned above, there is a morphological gap between *Hederorkis* and *Sirhookera/Adrorhizon*-complex of the organization of pollinaria. In all three genera, there are 4 pollinia gathered in two pairs, superposed or obliquely superposed. In *Hederorkis*, however, they are oblong-ellipsoid and hard, similar to those found in other vandoid genera, for example *Polystachya*. In both *Adrorhizon* and *Sirhookera* they are obliquely clavate, and easily becoming disintegrated, somewhat like in *Calanthe* or *Podochilus*. Additionally, their apices are transformed in granular, soft caudiculae. Caudiculae of *Hederorkis* are sticky, amorphous, like other vandoid genera.

Based on the morphological data and the results of the analyses of the *mat*K, *rpl*32-trnL, *rpl*16 intron and ITS sequences, it cannot be excluded that *Hederorkis* and *Adrorhizon/Sirhookera* share a common ancestor and the offspring evolved independently. One line leading to *Hederorkis* gained a vandoid type of pollinia, the other one, with *Adrorhizon/Sirhookera*, preserved the ancestral type. The *Adrorhizon/Sirhookera/Hederorkis* ancestor could have emerged in India from any *Bletilla*-like orchid and the plants could have spread to the Indian Ocean islands evolving and finally leading to the origin of *Hederokis*-like orchids.

*Bromheadia* Lindl. constitutes the lowest branch of the Vandeae/Polystachyinae/Adrorhizeae/*Hederorkis* clade. The genus has been classified within the monotypic subtribe proposed by Dressler [35], however due to its terminal inflorescence Szlachetko [9] included *Bromheadia* within Polystachyeae, although the genus, as well as *Collabium* Bl., *Diglyphosa* Bl. and *Claderia* Hook.f. are characterized by the possession of two pollinia instead of four found in *Polystachya* and other members of Polystachyinae. The morphological characters of *Bromheadia* preclude any closer relationship between this genus and the Adrorhizeae clade. In this study, due to the low bootstrap value (BS <50), as well as lack of such a clade (i.e. *Bromheadia*-Adrorhizeae-*Hederorkis*) in the strict consensus tree of the *mat*K gene and non-coding plastid regions, *Bromheadia* was not considered as part of Adrorhizeae, which has also been supported by earlier molecular analyses based on Xdh nuclear gene [36].

## Supporting Information

S1 Annex List of the DNA sequences used in the study. (DOC)

S2 Annex Combined plastid and nuclear ITS data matrix. (TXT)

S3 Annex Large data set for *mat*K analysis. (TXT)
