# Supplementary material for: Phylogenetic Placement and Taxonomy of the Genus Hederorkis (Orchidaceae)
Source: PLoS One. 2015 Apr 22;10(4):e0122306. doi: 10.1371/journal.pone.0122306 (PMC4406746; doi:10.1371/journal.pone.0122306)
Supplement: S1 Annex — (DOC) [file pone.0122306.s001.doc]

**Annex 1.**

| *Acampe sp.* | EF079278 | ex cult. Szlachetko s.n. |
| --- | --- | --- |
| *Acriopsis sp.* | EF065575 | Wien BG |
| *Aerides sp.* | EF079276 | ex cult. Szlachetko s.n. |
| *Altensteinia fimbriata* Kunth | EF065583 | Szlachetko s.n., Ecuador |
| Amesiella philippinensis (Ames) Garay | EF079281 | Heidelberg BG 104625 |
| *Ancistrochilus rothschildianus* O'Brien | EF079334 | Heidelberg BG 123168 |
| *Ancistrorhynchus paysanii* Senghas | EF079268 | Heidelberg BG 120000 |
| *Angraecopsis sp.* | EF079267 | Schoenbrunn |
| Angraecum eburneum Bory | EF065569 | ex cult. Szlachetko s.n. |
| Anguloa ruckeri Lindl. | EF065565 | ex cult. Szlachetko s.n. |
| *Ania hookeriana* (King & Pantl. ) Tang & F. T. Wang | EF079342 | Heidelberg BG 125230 |
| *Anneliesia candida* (Lindl.) Brieger & Lückel | EF079286 | Heidelberg BG 121311 |
| Ansellia africana Lindl. | EF079259 | Heidelberg BG 121891 |
| *Appendicula reflexa* Blume | EF079357 | Heidelberg BG 120017 |
| Arachnis flos-aeris Rchb.f. | EF079275 | Wien BG |
| *Arpophyllum giganteum* Hartw. ex Lindl. | EF065599 | ex cult. Szlachetko s.n. |
| *Artorima erubescens* (Lindl.) Dressler & G.E.Pollard | EF065587 | Heidelberg BG 121493 |
| *Arundina graminifolia* (D.Don) Hochr. | EF079333 | ex cult. Szlachetko s.n. |
| Ascocentropsis pusilla (Aver.) Senghas & Schildh. | EF079273 | Heidelberg BG 106770 |
| Aspasia lunata Lindl. | EF079200 | Heidelberg BG 120958 |
| *Aspidogyne pumila* (Cogn.) Garay | EF079297 | Szlachetko s.n., Ecuador |
| Barbosella dolichorhiza Schltr. | EF079328 | Heidelberg BG 123410 |
| *Barkeria spectabilis*  Bateman ex. Lindl. | EF079287 | Heidelberg BG 121293 |
| *Benthamia latifolia* A. Rich | EF079301 | Roberts DLR 10/03 / 19199 |
| *Bifrenaria harrisoniae* (Hook.) Rchb.f. | EF065567 | ex cult. Szlachetko s.n. |
| *Bletia purpurea* (Lam.) DC. | EF079335 | Heidelberg BG 108393 |
| *Bletilla striata* Rchb. f. | EF079331 | ex cult. Szlachetko s.n. |
| Bollea lawrenciana Rchb. f. | EF079239 | Ecuagenera |
| *Brachtia glumacea*  Rchb. f. | EF079201 | Heidelberg BG 123414 |
| *Brassia verrucosa* Lindl. | EF079203 | ex cult. Szlachetko s.n. |
| Brevilongium globuliferum (Kunth) Christenson | EF079197 | ex cult. Szlachetko s.n. |
| Bulbophyllum bequaertii De Wild. | EF065597 | Heidelberg BG 123231 |
| *Bulbophyllum macrobulbum* J.J. Sm. | EF065596 | Wien BG |
| Cadetia quinqueloba Schltr. | EF079346 | Heidelberg BG 120066 |
| Catasetum planiceps Lindl. | EF079266 | Hannover BG |
| *Catasetum sp.* | EF065571 | Brak informacji |
| *Cattleya leopoldii* Verschaff. ex Lem. | EF065586 | ex cult. Szlachetko s.n. |
| *Cattleya sp.* | EF079308 | Schoenbrunn |
| *Cephalantheropsis sp.* | EF079307 | Heidelberg BG 125239 |
| *Ceratostylis teres* Rchb. f. | EF079356 | Heidelberg BG 120023 |
| Chamaelorchis warscewiczii (Rchb. f.) Senghas & Lückel | EF065576 | Heidelberg BG 121294 |
| *Chiloglottis trapeziformis*  Fitzg. | EF065585 | ex. cult Melbourne / 0-569 |
| *Chloraea flavescens* | EF079299 | ex cult. Lueg s.n. |
| Chondrorhyncha andreae P.Ortiz | EF079237 | Heidelberg BG 103553 |
| *Christensonia vietnamica*  Haager | EF079272 | Heidelberg BG 120132 |
| Chrysocycnis schlimii Linden & Rchb.f. | EF079245 | Heidelberg BG 124831 |
| *Chysis bractescens*  Lindl. | EF079351 | Wien BG |
| *Cirrhopetalum umbellatum*  Linden | EF079343 | ex cult. Szlachetko s.n. |
| *Cischweinfia sp.* | EF079204 | Luis Mendoza s.n., Peru |
| *Coccineorchis sp.* | EF079293 | Ecuagenera |
| Cochlioda neozliana (Mast. ex L. Linden) Rolfe | EF079211 | Heidelberg BG 123978 |
| *Cochlioda rosea* (Lindl.) Benth. & Hook.f. | EF079212 | Hannover BG |
| *Coelia triptera* (Sm.) Steud. | EF079353 | Wien BG |
| *Coeliopsis hyacinthosma*  Rchb.f. | EF065564 | Heidelberg BG 121766 |
| *Coeloglossum viride*  Hartm. | EF079300 | Szlachetko s.n., Poland |
| *Coelogyne barbata*  Lindl. ex Griff. | EF079332 | Schoenbrunn |
| *Collare-Stuartense multistellare* (Rchb.f.) Senghas & Bockemühl | EF079213 | Heidelberg BG 123846 |
| Comparettia speciosa Rchb. f. | EF079191 | ex cult. Szlachetko s.n. |
| *Constantia cipoensis*  Porto & Brade | EF079322 | Heidelberg BG 105289 |
| *Crepidium sp.* | EF079338 | Hamburg BG 602 B 337 |
| *Cuitlauzina pendula* La Llave & Lex. | EF079218 | ex cult. Szlachetko s.n. |
| *Cyclopogon lindleyanus*  Schltr. | EF079289 | Wien BG |
| *Cyclopogon pamii* (Braid) Mansf. & Herter | EF079290 | Popov cult. 99/602 |
| *Cycnoches sp.* | EF079264 | Schoenbrunn |
| *Cydoniorchis tetragona* (Lindl.) Senghas | EF079250 | Heidelberg BG 122447 |
| *Cynorkis grandiflora*  Ridl. | EF065584 | ex cult. Szlachetko s.n. |
| *Cyrtidiorchis frontinoensis* (Garay) Rauschert | EF079246 | Ecuagenera |
| Cyrtochilum macranthum Kraenzl. | EF079205 | Szlachetko s.n., Ecuador |
| *Cyrtopodium andersonii* (Lamb. ex Andrews) R.Br. in W.T.Aiton | EF079263 | Szlachetko s.n., French Guyana |
| *Dendrobium findleyanum* C.S.P.Parish & Rchb.f. | EF079348 | ex cult. Szlachetko s.n. |
| *Dendrochilum scriptum* Carr | EF079347 | Heidelberg BG 121779 |
| *Dichaea caveroi* D.E.Benn. & Christenson | EF079244 | Heidelberg BG 124022 |
| *Dienia latifolia* (Sm.) M.A.Clem. & D.L.Jones | EF079340 | Hamburg BG 339 |
| *Dinema cubincola* (Borhidi) H.Dietr. | EF079321 | Heidelberg BG 103394 |
| *Diplocaulobium aureicolor* (J.J.Sm.) A.D.Hawkes | EF079344 | ex cult. Szlachetko s.n. |
| *Diplocaulobium validicolle* Kraenzl. | EF079345 | Heidelberg BG 123424 |
| *Dipodium paludosum* Rchb .f. | EF079261 | Heidelberg BG 122008 |
| Doritis pulcherrima Lindl. | EF079282 | Heidelberg BG 125529 |
| *Dressleria dilecta* (Rchb.f.) Dodson | EF079265 | Heidelberg BG 122837 |
| *Dryadella hirtzii* Luer | EF079327 | Heidelberg BG 123364 |
| *Earina autumnalis* Hook. f. | EF079336 | Heidelberg BG 124446 |
| *Earina deplanchei* Rchb. f. | EF079337 | Heidelberg BG 120097 |
| *Echioglossum sp.* | EF065574 | Heidelberg BG 123029 |
| Elleanthus caravata Rchb. f. | EF079358 | Szlachetko s.n., French Guyana |
| *Elleanthus sp.* | EF079359 | Szlachetko s.n., Ecuador |
| *Eltroplectris roseoalba* (Rchb.f.) Hamer & Garay | EF065578 | Hannover BG |
| *Encyclia atropurpurea* Schltr. | EF079318 | ex cult. Szlachetko s.n. |
| *Epidendrum rigidum* Jacq. | EF079311 | Wien BG |
| *Epistephium* sp. | EF065602 | Szlachetko s.n., Ecuador |
| *Eria javanica* (Sw.) Blume | EF079354 | ex cult. Szlachetko s.n. |
| *Eriopsis sceptrum* Rchb.f. & Warsz. | EF079253 | Ecuador |
| *Eulophia petersii* Rchb. f. | EF079257 | Schoenbrunn |
| Eulophia streptopetala Lindl. | EF079258 | Schoenbrunn |
| *Eurystyles cotyledon* Wawra | EF079294 | Ecuagenera |
| *Eurystyles sp.* | EF079295 | Szlachetko s.n., Ecuador |
| *Flickingeria insularis* Seidenf. | EF079349 | Heidelberg BG 120613 |
| *Frondaria sp.* | EF079324 | Szlachetko s.n., Ecuador |
| *Gastrorchis pulchra* Humbert & H.Perrier | EF079305 | Heidelberg BG 104634 |
| *Gomesa planifolia* Klotzsch ex Rchb.f. | EF079196 | ex cult. Szlachetko s.n. |
| Gongora galeata Rchb. f. | EF079251 | ex cult. Szlachetko s.n. |
| *Grammatophyllum papuanum* J. J. Sm. | EF079262 | Heidelberg BG 122847 |
| *Hapalochilus nitidus* (Schltr.) Senghas | EF065598 | Heidelberg BG 123418 |
| *Helcia brevis* (Rolfe) Dodson | EF079229 | Hannover BG |
| *Houlletia clarae* Schltr. | EF065562 | Heidelberg BG 125504 |
| *Ida hirtzii* (Dodson) A.Ryan & Oakeley | EF065566 | Ecuagenera |
| *Isabelia virginalis* Barb.Rodr. | EF079320 | Heidelberg BG 120030 |
| *Isochilus sp.* | EF079350 | ex cult. Szlachetko s.n. |
| *Jacquiniella globosa* (Jacq.) Schltr. | EF065588 | Heidelberg BG 120088 |
| *Jumellea sp.* | EF065570 | Schoenbrunn |
| Kegeliella atropilosa L.O.Williams & A.H.Heller | EF079232 | Heidelberg BG 122428 |
| *Lacaena spectabilis* Rchb. f. | EF079233 | Heidelberg BG 122338 |
| *Laelia gouldiana* Rchb. f. | EF079315 | ex cult. Szlachetko s.n. |
| *Lankesterella gnomus* (Kraenzl.) Hoehne | EF065581 | Takase s.n., Brasil |
| *Lankesterella* sp. | EF065582 | Ecuador |
| *Lemboglossum bictoniense* (Bateman) Halb. ex Christenson | EF079198 | ex cult. Szlachetko s.n. |
| *Leochilus carinatus* (Knowles & Westc.) Lindl. | EF079194 | Heidelberg BG 106230 |
| Leptotes bicolor Lindl. | EF079319 | Heidelberg BG 121368 |
| Liparis nervosa (Thunb.) Lindl. | EF065594 | B339 |
| *Lockhartia amoena* Endrés & Rchb.f. | EF065560 | Heidelberg BG 121793 |
| Lophiaris lanceana (Lindl.) Braem | EF079283 | Heidelberg BG 122621 |
| *Lycaste locusta* Rchb. f. | EF079254 | Ecuagenera |
| Macroclinium alleniorum Dressler & Pupulin | EF079188 | Heidelberg BG 121532 |
| *Malaxis monophyllos* (L.) Sw. | EF065593 | H. Presser , Germany |
| *Maxillaria punctata* Lodd. | EF079249 | Ecuagenera |
| *Maxillaria variabilis* Bateman ex Lindl. | EF079247 | Ecuagenera |
| *Mediocalcar decoratum* Schuit. | EF079355 | Heidelberg BG 123319 |
| Meiracyllium trinasutum Rchb. f. | EF079317 | Heidelberg BG 120031 |
| *Mesadenella cuspidata* (Lindl.) Garay | EF079296 | Munich BG |
| Mesoglossum londesboroughianum (Rchb.f.) Halb. | EF065561 | Heidelberg BG 122882 |
| Mexicoa ghiesbreghtiana (A.Rich. & Galeotti) Garay | EF079209 | Heidelberg BG 123603 |
| Microterangis hariotiana (Schltr.) Senghas | EF079271 | Heidelberg BG 122893 |
| *Miltonioides sp.* | EF079210 | Heidelberg BG 124450 |
| Monophyllorchis maculata Garay | EF065603 | Ecuagenera |
| *Mormodes andreettae* Dodson | EF065563 | Ecuagenera |
| *Mormodes vinacea* (Lindl.) Schltr. | EF079252 | Heidelberg BG 122384 |
| *Mormolyca ringens* (Lindl.) Schltr. | EF079270 | Schoenbrunn |
| *Myoxanthus serripetalus* (Kraenzl.) Luer | EF065600 | Heidelberg BG 124228 |
| *Nanodes medusae* Rchb. f. | EF079313 | Szlachetko s.n., Ecuador |
| *Nanodes schlechterianum* Brieger | EF079314 | Heidelberg BG 121366 |
| *Neobenthamia sp.* | EF079288 | Heidelberg BG s.n. |
| *Neodryas weberbaueriana* (Kraenzl.) Schltr. | EF079206 | Ecuagenera |
| *Neomoorea irrorata* Rolfe | EF079256 | Schoenbrunn |
| Neottia nidus-avis (L.) Rich. | EF079303 | Fischer 39 / 20650 |
| Notylia venezuelana Schltr. | EF079193 | ex cult. Szlachetko s.n. |
| *Oberonia sp.* | EF079339 | Heidelberg BG 120006 |
| *Octomeria sp.* | EF079352 | Heidelberg BG 125079 |
| Odontoglossum harryanum Rchb. f. | EF079216 | Ecuagenera |
| *Oeceoclades rauhii* (Senghas) Garay & P.Taylor | EF079260 | Heidelberg BG 108020 |
| *Oerstedella centradenia* Rchb. f. | EF079312 | Heidelberg BG 101762 |
| *Oliveriana brevilabia* (C.Schweinf.) Dressler & N.H.Williams | EF079202 | Heidelberg BG 123415 |
| *Oncidium orthotis* Rchb.f. | EF079208 | Ecuagenera |
| *Ornithocephalus sp.* | EF079226 | Szlachetko s.n., Ecuador |
| *Osmoglossum convallarioides* Schltr. | EF079219 | Heidelberg BG 123838 |
| *Palmorchis sp.* | EF079361 | Salazar 6216/16446 |
| *Palumbina candida* Rchb. f. | EF079217 | Heidelberg BG 124275 |
| *Paphinia seegeri* G.Gerlach | EF079236 | Heidelberg BG 122881 |
| *Pelexia sp.* | EF079291 | Ecuagenera |
| *Pescatoria lehmannii* Rchb. f. | EF079240 | Ecuagenera |
| Phaius tancarvilleae *(L'Hér.) Blume* | EF079306 | ex cult. Szlachetko s.n. |
| *Phymatidium sp.* | EF079227 | ex cult. Szlachetko s.n. |
| *Physothallis harlingii* Garay | EF065591 | Heidelberg BG 124671 |
| *Platystele stenostachya* (Rchb.f.) Garay | EF079326 | ex cult. Szlachetko s.n. |
| Plectrelminthus caudatus (Lindl.) Summerh. | EF079269 | Schoenbrunn |
| *Pleurothallis josephensis*  Barb. Rodr. | EF079330 | Heidelberg BG 120676 |
| *Polycycnis sp.* | EF079231 | Ecuagenera |
| *Polyrrhiza funalis* Pfitzer | EF079285 | Heidelberg BG 122836 |
| *Polystachya ottoniana* Rchb. f. | EF065577 | Hannover BG |
| *Ponera sp.* | EF079323 | Heidelberg BG 121987 |
| *Pseudolaelia vellozicola* (Hoehne) Porto & Brade | EF065589 | Heidelberg BG 121730 |
| *Psygmorchis pusilla* (L.) Dodson & Dressler | EF079222 | Ecuagenera |
| *Pteroceras sp.* | EF079279 | ex cult. Szlachetko s.n. |
| *Pterostylis curta* R.Br. | EF079298 | ex cult. Szlachetko s.n. |
| *Rangaeris sp.* | EF079277 | Heidelberg BG 121559 |
| *Raycadenco sp.* | EF079223 | Ecuagenera |
| *Restrepia trichoglottis* Hort. ex Gentil | EF079329 | Heidelberg BG 123495 |
| *Rhipidoglossum ruttilum* Schltr. | EF065573 | Heidelberg BG 122993 |
| *Rhyncholaelia digbyana* (Lindl.) Schltr. | EF079309 | ex cult. Szlachetko s.n. |
| *Rhynchopera sp.* | EF065590 | Ecuagenera |
| *Rodriguezia decora* Rchb. f. | EF079199 | Heidelberg BG 120038 |
| *Rossioglossum schlieperianum* (Rchb.f.) Garay & G.C.Kenn. | EF079221 | Schoenbrunn |
| *Rusbyella* sp. | EF079207 | Ecuagenera |
| *Sarcochilus japonicus* Miq. | EF079274 | Heidelberg BG 122933 |
| *Sarcoglottis neglecta* Christenson | EF079292 | GUF 99-022 |
| *Sarcoglyphis thailandica* Seidenf. | EF079284 | Heidelberg BG 120021 |
| *Sauroglossum elatum* Lindl. | EF065580 | Hamburg BG |
| *Scaphosepalum ursinum* Luer | EF079325 | Heidelberg BG 124283 |
| *Scelochilus* sp. | EF079192 | Luis Mendoza, Peru |
| *Schomburgkia sp.* | EF079316 | ex cult. Szlachetko s.n. |
| *Seegeriella pinifolia* Senghas | EF079189 | Heidelberg BG 103775 |
| *Selenipedium aequinoctiale* Garay | EF079360 | Ecuagenera |
| Senghasia parvilabris (Schltr.) Szlach. | EF079238 | Heidelberg BG 121377 |
| Sieviekingia reichenbachia Kraenzl. | EF079234 | Heidelberg BG 122825 |
| Sigmatostalix buchtienii Kraenzl. | EF079215 | Heidelberg BG 121360 |
| *Sirhookera lanceolata* Kuntze | EF079341 | Heidelberg BG 120208 |
| *Skeptrostachys sp.* | EF065579 | Takase s.n., Brazil |
| *Sobralia rosea* Poepp. & Endl. | EF065601 | Szlachetko s.n., Ecuador |
| *Solenidiopsis tigroides* (C.Schweinf.) Senghas | EF079214 | Heidelberg BG s.n. |
| *Sophronitis cernua* (Lindl.) Lindl. | EF079310 | Heidelberg BG 120065 |
| *Stenia bismarckii* Dodson & D.E.Benn. | EF079241 | ex cult. Szlachetko s.n. |
| *Stictophyllorchis* sp*.* | EF079190 | Ecuagenera |
| *Telipogon sp.* | EF079228 | Heidelberg BG 108219 |
| Thelymitra campanulata Lindl. | EF079302 | Corrigen Airstrip WA / O-843 |
| *Thrixspermum arachnites* Rchb. f. | EF065572 | Heidelberg BG 104401 |
| *Thysanoglossa jordanensis* Porto & Brade | EF079224 | Heidelberg BG 120057 |
| *Ticoglossum krameri* (Rchb.f.) Lucas Rodr. ex Halb. | EF079220 | Ecuagenera |
| *Trevoria escobariana* Garay | EF079235 | Ecuagenera |
| Trias intermedia Seidenf. & Smitinand | EF065595 | Heidelberg BG 122330 |
| *Trichoglottis cirrhifera* Teijsm. & Binn. | EF079280 | Heidelberg BG 103795 |
| *Trichopilia sp.* | EF079230 | Luis Mendoza, Peru |
| *Trigonidium grande* Garay | EF079248 | Ecuagenera |
| *Trisetella triglochin* (Rchb. f.) Luer | EF065592 | Heidelberg BG 103324 |
| Tropidia graminea Blume | EF079304 | Duangjai 040 / 21775 |
| *Vandopsis parishii* Schltr. | EF065568 | Schoenbrunn |
| *Xylobium squalens* Lindl. | EF079255 | ex cult. Szlachetko s.n. |
| *Zelenkoa onusta* (Lindl.) M.W.Chase & N.H.Williams | EF079195 | ex cult. Szlachetko s.n. |
| Zygopetalum mackaii Hook. | EF079243 | ex cult. Szlachetko s.n. |
| *Zygopetalum maxillare* Lood. | EF079242 | Heidelberg BG 124740 |
| Zygostates alleniana Kraenzl. | EF079225 | Heidelberg BG 108182 |
